# The Anti-Non-Small Cell Lung Cancer Cell Activity by a mTOR Kinase Inhibitor PQR620

**DOI:** 10.3389/fonc.2021.669518

**Published:** 2021-06-10

**Authors:** Jian-hua Zha, Ying-chen Xia, Chun-lin Ye, Zhi Hu, Qin Zhang, Han Xiao, Ben-tong Yu, Wei-hua Xu, Guo-qiu Xu

**Affiliations:** ^1^ Department of Thoracic Surgery, The First Affiliated Hospital of Nanchang University, Nanchang, China; ^2^ Department of Respiratory Medicine, Suzhou Hospital Affiliated Nanjing Medical University, Suzhou, China; ^3^ Department of Thoracic Surgery, Union Hospital, Tongji Medical College, Huazhong University of Science and Technology, Wuhan, China; ^4^ Department of Cardiothoracic Surgery, The Second Affiliated Hospital of Soochow University, Suzhou, China

**Keywords:** non-small-cell lung carcinoma, mammalian target of rapamycin, Akt, PQR620, signalings

## Abstract

In non-small-cell lung carcinoma (NSCLC), aberrant activation of mammalian target of rapamycin (mTOR) contributes to tumorigenesis and cancer progression. PQR620 is a novel and highly-potent mTOR kinase inhibitor. We here tested its potential activity in NSCLC cells. In primary human NSCLC cells and established cell lines (A549 and NCI-H1944), PQR620 inhibited cell growth, proliferation, and cell cycle progression, as well as cell migration and invasion, while inducing significant apoptosis activation. PQR620 disrupted assembles of mTOR complex 1 (mTOR-Raptor) and mTOR complex 2 (mTOR-Rictor-Sin1), and blocked Akt, S6K1, and S6 phosphorylations in NSCLC cells. Restoring Akt-mTOR activation by a constitutively-active Akt1 (S473D) only partially inhibited PQR620-induced cytotoxicity in NSCLC cells. PQR620 was yet cytotoxic in Akt1/2-silenced NSCLC cells, supporting the existence of Akt-mTOR-independent mechanisms. Indeed, PQR620 induced sphingosine kinase 1 (SphK1) inhibition, ceramide production and oxidative stress in primary NSCLC cells. *In vivo* studies demonstrated that daily oral administration of a single dose of PQR620 potently inhibited primary NSCLC xenograft growth in severe combined immune deficient mice. In PQR620-treated xenograft tissues, Akt-mTOR inactivation, apoptosis induction, SphK1 inhibition and oxidative stress were detected. In conclusion, PQR620 exerted potent anti-NSCLC cell activity *via* mTOR-dependent and -independent mechanisms.

## Introduction

Lung cancer is a leading cause of cancer-related human mortalities worldwide (1, 2). Non-small-cell lung carcinoma (NSCLC) accounts for over 85% of all lung cancers and is relatively insensitive to chemotherapy (3, 4). Currently, NSCLC (especially early-stage cancers) are primarily treated by surgical resection, though chemotherapy has been utilized increasingly both pre-operatively (neo-adjuvant chemotherapy) and post-operatively (adjuvant chemotherapy) (3, 4). Yet, NSCLC has one of the worst prognosis among all human malignancies (1, 2) with only 8-10 months of average survival for advanced NSCLC patients (1, 2). Therefore, there is an urgent need to explore novel and more efficient molecular-targeted therapies against this devastating disease (3, 4).

The serine/threonine protein kinase mammalian target of rapamycin (mTOR) is a key component of two protein complexes, mTOR complex 1 (mTORC1) and mTOR complex 2 (mTORC2) (5–9). Activation of mTOR is essential for a number of core cellular behaviors, including cell growth, proliferation and motility, survival, as well as protein synthesis, autophagy inhibition, apoptosis-resistance and transcription (5–9). Dysregulation and aberrant activation of mTOR is commonly detected in NSCLC, which is associated with tumorigenesis and cancer progression (5, 6). mTOR is a validate and important therapeutic target of NSCLC (5, 6).

The first generation of mTOR inhibitors, including rapamycin and its analogs, can only partially inhibit mTORC1 and had limited effect on mTORC2 (7, 10). Moreover, mTOC1 inhibition in cancer cells is able to induce feedback activation of multiple oncogenic signaling pathways, including Akt and Erk-MAPK, which compromise their anti-cancer activities (11, 12). In addition, rapamycin and its analogs often have poor water solubility (7, 10). Therefore, the second generation of mTOR inhibitors, or mTOR kinase inhibitors, is developed (13, 14). These inhibitors simultaneously block mTORC1 and mTORC2 without inducing feedback activation of oncogenic signaling (13, 14). Several mTOR kinase inhibitors have displayed profound anti-NSCLC cell activity (5, 15).

Recent studies have developed PQR620 as a highly potent mTOR kinase inhibitor that crosses the blood-brain barriers (16–18). It has displayed fine selectivity for mTOR over other protein kinases (19). PQR620 showed excellent pharmacokinetics, as it reached maximum concentration in plasma and brain within 30 min after injection in mice (19) with a half-life of over 5 h (19). Daily oral administration of PQR620 in mice can potently inhibit ovarian carcinoma xenograft growth (16, 19). It has also demonstrated anti-tumor activity in lymphomas either alone or in combination with venetoclax (18). Its potential effect on NSCLC cells and underlying mechanisms are tested here.

## Materials and Methods

### Chemicals and Reagents

JC-1, EdU (5-Ethynyl-2′-deoxyuridine), DAPI (4′,6-diamidino-2-phenylindole), TUNEL (Terminal deoxynucleotidyl transferase dUTP nick end labeling) and CellROX fluorescence dyes, as well as Annexin V and propidium iodide (PI) were purchased from Thermo-Fisher Invitrogen Co. (Shanghai, China). Antibodies of p-Akt (Ser 473, #9271), Akt1 (#2938), p70 S6 Kinase (S6K1 #9202), p-S6K1 (Thr389, #9205), p-S6 (Ser235/236, #2211), S6 (#2317), p-4E-BP1 (Ser65), mTOR pathway antibody sampler kit (#9964), cleaved caspase antibody sampler Kit (#9929), Erk1/2 (#4695), p-Erk1/2 (9101), GAPDH (glyceraldehyde-3-phosphate dehydrogenase, #5174), SphK1 (#12071), and β-tubulin (#2146) were provided by Cell Signaling Technologies (Beverly, MA). Fetal bovine serum (FBS), antibiotics and other cell culture reagents were obtained from Hyclone(Logan, UT). From Sigma-Aldrich chemicals (St. Louis, Mo), puromycin, polybrene, z-DEVD-fmk, z-VAD-fmk, N-acetylcysteine (NAC), perifosine, MK-2206, INK-128, and AZD-2014 were obtained. Sphingosine 1-phosphate (S1P) powder was purchased from Sigma-Aldrich (73914) and was dissolved methanol-BSA solution to achieve 250 μM stock solution according to the attached protocol. PQR620 was from MedChemExpress (Beijing, China). For *in vitro* experiments, PQR620 was dissolved in DMSO to make 20 mM stock solution, the latter was added to cell medium with the final DMSO concentration at 0.1% (vehicle). Cell Counting Kit-8 (CCK-8) assay kit was provided by Beyotime (Suzhou, China).

### Cells

Established NSCLC cell lines, A549 and NCI-H1944, as well as BEAS-2B lung (bronchial) epithelial cells were provided by Shanghai Institute for Biological Sciences (Shanghai, China). Cells were cultured according to the supplier’s instructions. Primary NSCLC cells, derived from three primary NSCLC patients (pNSCLC-1, pNSCLC-2 and pNSCLC-3, all with PTEN depletion), as well as the primary lung epithelial cells, were provided by Dr. Jiang (20, 21). The primary human NSCLC cells were cultured in high glucose (25 mmol/L) DMEM/F-12 growth medium with 12% FBS plus EGF (2.5 ng/mL) and insulin (2.5 ng/mL) in culture flasks (3,0000-50,000 cells per flask). Cells were passed for 8-10 generations. Protocols were reviewed and approved by the Ethics Committee of Nanchang University (NU-BMS-1805037), and were conformed to the guidelines of the 2000 Helsinki declaration. Written-informed consent was obtained from all subjects before their participation. Cells were routinely subjected to mycoplasma and microbial contamination examination. To verify cell genotypes, short-tandem repeat profiling, population doubling time, and cell morphology were always checked.

### Colony Formation

For colony formation assay, NSCLC cells at 1 × 10^4^ cells per well, were initially seeded into 10-cm tissue-culturing dishes, and maintained under PQR620-containing medium (with 10% FBS). After ten days, colonies were fixed, stained and manually counted.

### Cell Cycle Studies

In brief, following the applied PQR620 treatment, NSCLC cells were washed, trypsinized and re-suspended in 95% ethanol solution. Thereafter, cells were centrifuged and resuspended in 1 mL of PI staining solution. Cell cycle distribution was studied by a FACS-calibur flow cytometry (Beckman-Coulter, Shanghai, China).

### Western Blotting

Cell and tissue lysate samples were achieved by using commercial lysis buffer (Biyuntian Co, Wuxi, China). Quantified protein lysates (40 µg per treatment into each lane) were separated on 10% to 12% SDS-PAGE gels and transferred to nitrocellulose filter membranes. The membranes were blocked and incubated with applied primary antibodies (at 4°C overnight), followed by incubation with HRP-conjugated secondary antibodies (1 h at room temperature). The targeted protein bands were visualized under ChemiScope 3300 Mini (Clinx) *via* ECL substrates (Invitrogen, Shanghai, China).

### Co-Immunoprecipitation (Co-IP)

For each treatment, protein lysates (1,000 μg per treatment) were pre-cleared and then incubated with anti-mTOR antibody (Santa Cruz Biotech) for 16 h. Afterward, the protein A/G Sepharose (“Beads”, 30 µl per treatment) was added back to the lysates. mTOR-immunoprecipitated proteins were tested by Western blotting assays.

### Quantitative Real-Time Reverse Transcriptase Polymerase Chain Reaction (qPCR)

Following treatment, TRIzol reagents were utilized to extract total RNA and qPCR was performed using the described protocol (22). *SphK1* and *GAPDH* mRNA primers were purchased from OriGene (Shanghai, China).

### EdU Assays

A Cell-Light EdU (5-ethynyl-2′-deoxyuridine) Apollo 567 Kit (Ribobio, Guangzhou, China) was carried out to quantify cell proliferation. Briefly, NSCLC cells were seeded into 96-well plates at 5×10^3^ cells/well. After 48 h, 100 μl medium containing 10 μM EdU was added into each well. Cells were incubated for 2 h, fixed, and co-stained with DAPI. EdU, and DAPI staining was captured by a fluorescence microscopy (Nikon, Japan). For each treatment, five random views with a total of 1,000 nuclei were included to calculate the average EdU ratio (% vs. DAPI).

### CCK-8 Assay

Briefly, cells with applied treatment were seeded into 96-well plates at 5 × 10^3^ cells/well. After treatment, 10 μl CCK-8 solution was added to each well and incubated for 2 h. In each well, CCK-8 optical density (OD) was measured at 450 nm.

### Trypan Blue Assaying of Cell Death

NSCLC cells were seeded into six-well plates and were treated as described. Trypan blue dye was added to stain the “dead” cells, with its ratio was determined using an automated cell counter (Merck Millipore, Shanghai, China).

### Apoptotic Nuclei Assays

NSCLC cells were seeded into 96-well plates at 5 × 10^3^ cells/well. After the applied treatment, cell nuclei were co-stained with TUNEL and Hoechst-33342. The apoptotic nuclei displayed condensed/fragmented Hoechst-33342 staining, and some cells were positive for TUNEL staining. For each treatment, five random views with a total of 1,000 nuclei were included to calculate the average apoptotic nuclei ratio.

### Annexin V FACS

A FITC Annexin V Apoptosis Detection Kit I (BD Biosciences) was utilized. Briefly, NSCLC cells with applied treatments were harvested, washed and resuspended in 1× Binding Buffer (1×10^6^ cells/ml). Thereafter, 5 μl PI and 5 μl Annexin V were added. Cells were further incubated for 15 min and were analyzed *via* a FACSCalibur Flow Cytometer (BD Biosciences).

### Mitochondrial Depolarization

In apoptotic cells with mitochondrial depolarization, JC-1 fluorescence dye is able to aggregate mitochondria to form green monomers (23). Following treatment, NSCLC cells were stained with JC-1 (15 μg/ml, Sigma), washed, and examined under a fluorescence spectrofluorometer (F-7000, Hitachi, Japan) at 488 nm (green). The representative JC-1 fluorescence images integrating green (at 488 nm, mitochondrial depolarization) and red (at 625 nm, normal mitochondrial membrane potential) fluorescence channels were presented.

### Transwell Assays

Transwell assay was performed using 12.0 μm Transwell Permeable Supports (Corning, Shanghai, China) using described protocols (24, 25). Five random views of each condition were included to calculate the average number of migrated/invaded cells.

### Single-Strand DNA (ssDNA) Detection

NSCLC cells with applied treatments were seeded into 96-well plates at 5 × 10^3^ cells/well. After the applied treatment, DNA break intensity was tested by a ssDNA apoptosis ELISA kit (Merck Millipore, Shanghai, China). ELISA absorbance was tested at 405 nm in each well.

### Constitutively Active Mutant Akt1

A recombinant adenoviral construct encoding the constitutively-active Akt1 (caAkt1, S473D) was provided by Dr. Li at Wenzhou Medical University (26, 27), and was transduced to pNSCLC-1 cells. Cells with GFP were then sorted by FACS and monoclonal single cells were distributed into 192-well plates. In stable cells, caAkt1 expression was verified by Western blotting.

### Akt1/2 shRNA

Akt1/2 shRNA lentiviral particles (sc-37030-V) were provided by Santa Cruz Biotech and were added directly to primary NSCLC cells. After 24 h, puromycin (5.0 μg/ml) was added to select stable cells, where over 95% Akt1/2 protein knockdown efficiency was achieved.

### Lipid Peroxidation Assay

NSCLC cells were seeded into six-well plates (8 ×10,000 cells per well) and were subjected to applied treatments. We performed a thiobarbituric acid reactive substances (TBAR) activity assay to quantify cellular lipid peroxidation levels using a described protocol (28, 29).

### Assaying of Reactive Oxygen Species (ROS) Contents

NSCLC cells were seeded into six-well plates and were treated with PQR620. Cells were then stained with CellROX (5 μg/ml), washed, and tested under a spectrofluorometer (F-7000, Hitachi, Japan) at 625 nm (red fluorescence). The representative CellROX fluorescence images were presented as well.

### Assaying of Sphingosine Kinase 1 (SphK1) Activity and Ceramide Contents

NSCLC cells were seeded into six-well plates (8 ×10,000 cells per well) and were subjected to applied treatments. The protocols for testing SphK1 activity by measuring radio-labeled sphingosine-1-phosphate (S1P) spots were described elsewhere (30). SphK1 activity was expressed as pmol/h/g protein. By using the described protocol (31), cellular ceramide contents were examined and expressed in fmol by nmol of phospholipids.

### Tumor Xenograft Studies

Six-week-old severe combined immunodeficient (SCID) mice (half male and half female, 18.2–19.2 g) were maintained under Animal Facility of Suzhou University (Suzhou, China). A549 cells or pNSCLC-1 cells were subcutaneously (*s.c.*) injected to the right flanks of SCID mice at 3 × 10^6^ per mouse. When xenograft tumors were established and tumor volume reached 100 mm^3^, it was labeled as “Day-0.” NSCLC xenograft-bearing SCID mice were randomly assigned into two groups, receiving either vehicle control or PQR620 administration. At the time of drug administration, PQR620 (25 mg) was fresh dissolved in DMSO (0.5 ml). Afterward, 20% HP-β-CD (hydroxypropyl-β-cyclodextrin/water, 4.5 ml) was added, and the mixture was vortexed and sonicated to form homogeneous solution. The solution was given to SCID mice by oral gavage. The DMSO-HP-β-CD solution was administrated as vehicle control (19). Tumor dimensions were measured and tumor volume was estimated as per: V = length × width × height × 0.5236. All animal experiments were approved by Animal Ethics Board of Nanchang University (NU-BMS-1805037).

### Statistical Analysis

Data were presented as mean ± standard deviation (SD). Statistical analyses were carried out using one-way analysis of variance (ANOVA) followed by Tukey’s multiple comparison test (GraphPad Prism 5.01). The Student t test (Excel2007) was applied to compare statistical difference between two groups. *p* < 0.05 was considered as statistically significant.

## Results

### PQR620 Exerts Robust Anti-NSCLC Cell Activity

**Figure 1:**
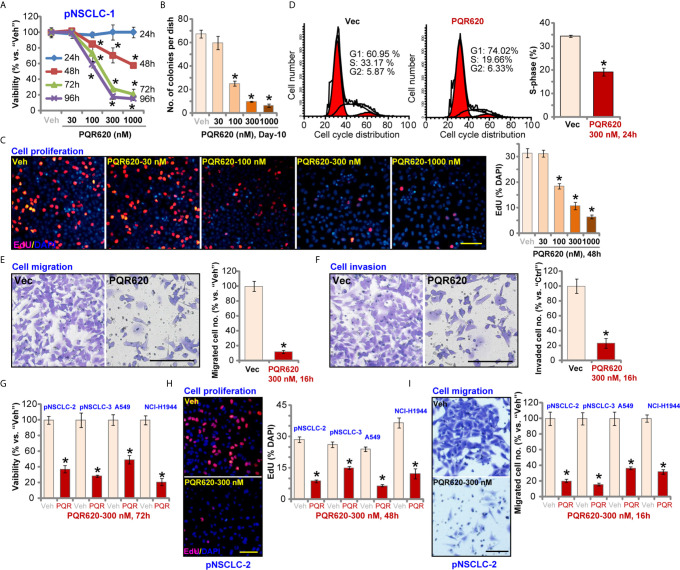
PQR620 exerts robust anti-NSCLC cell activity. Primary NSCLC cells that were derived from three different patients (pNSCLC-1/-2/-3) and established cell lines (A549 and NCI-H1944) were treated with PQR620 (at applied concentrations) or vehicle control (“Veh”). Cell viability (CCK-8 OD, **A**, **G**), colony formation (results were quantified, **B**), cell proliferation (EdU incorporation, **C**, **H**), cell cycle progression (PI-FACS, **D**), cell migration **(E, I)** and invasion **(F)** were tested by the described assays. Data were presented as mean ± standard deviation (SD, n=5). *p< 0.05 vs. “Veh” cells. Experiments were repeated five times with similar results. Scale bar= 100 μm **(C, E, F, H, I)**.

Primary human NSCLC cells, pNSCLC-1, were cultured in FBS-containing medium and treated with PQR620 (at 30-1000 nM). CCK-8 assays were carried out to test cell viability after applied time periods. As shown, PQR620, in a concentration-dependent manner, decreased viability (CCK-8 OD) in pNSCLC-1 cells **(** **Figure 1A**). Viability reduction by PQR620 was significant at 100-1000 nM, but not at 30 nM (**Figure 1A**). In addition, PQR620 displayed a time-dependent response in inhibiting pNSCLC-1 cell viability and required at least 48 h to obtain a significant effect (**Figure 1A**). Colony formation assay results in **Figure 1B** demonstrated that PQR620 (100–1000 nM) significantly inhibited pNSCLC-1 cell colony formation. It was again ineffective at 30 nM (**Figure 1B**). To test cell proliferation, EdU-nuclei staining assays were performed. Results showed that in pNSCLC-1 cells, PQR620 dose-dependently decreased EdU-positive nuclei ratio (**Figure 1C**). EdU ratio (% vs. DAPI) reduction was significant after 100 to 1,000 nM of PQR620 treatment (**Figure 1C**). As shown, 300 nM of PQR620 displayed significant effect in viability, colony formation, and proliferation assays (**Figures 1A–C**). This concentration was close to IC-50 and was therefore selected for further studies.

To test cell cycle progression, PI-FACS assays were performed. Results in **Figure 1D** showed that PQR620 (300 nM, 48 h) treatment resulted in decreased S-phase cells, but there was an increase in the number of G1-phase cells, suggesting that PQR620 induced G1-S arrest in pNSCLC-1 cells. Cell migration was tested using Transwell assays, and we found that pNSCLC-1 cell migration was potently inhibited by PQR620 (300 nM, 16 h) treatment (**Figure 1E**). *In vitro* pNSCLC-1 cell invasion, tested by Matrigel Transwell assays, was suppressed by same PQR620 treatment (**Figure 1F**).

We also tested the potential effect of PQR620 in other NSCLC cells. The primary NSCLC cells-derived from two other patients, pNSCLC-2 and pNSCLC-3, as well as established cell lines (A549 and NCI-H1944) were cultured, and treated with PQR620 (300 nM). As shown, PQR620 resulted in robust viability (CCK-8 OD) reduction (**Figure 1G**), proliferation inhibition (EdU-positive nuclei ratio reduction, **Figure 1H**) and migration inhibition (**Figure 1I**). Therefore, in primary and established NSCLC cells, PQR620 potently inhibited cell viability, proliferation and cell cycle progression, as well as cell migration and invasion.

### PQR620 Provokes Apoptosis Activation in NSCLC Cells

**Figure 2:**
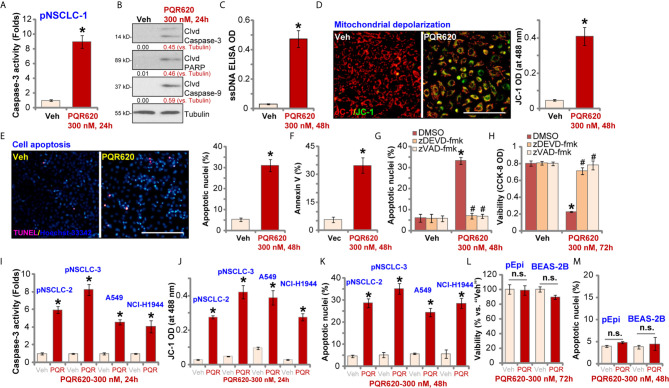
PQR620 provokes apoptosis activation in NSCLC cells. Primary NSCLC cells were derived from three different patients (pNSCLC-1/-2/-3). Established NSCLC cell lines (A549 and NCI-H1944), human lung epithelial cells (“pEpi”), or BEAS-2B cells were treated with PQR620 (300 nM) or vehicle control (“Veh”). Cells were further cultured for indicated time periods, caspase activation **(A**, **B**, **I)**, single strand DNA contents (ELISA assays, **C**), mitochondrial depolarization (JC-1 green monomers accumulation, **D**, **J**) were tested. Cell apoptosis was tested by Hoechst-33342 apoptotic nuclei staining assays **(E**, **K**, **M)** and Annexin V-FACS assays **(F)**, with cell viability tested by CCK-8 assays **(L)**. pNSCLC-1 cells were pretreated for 30 min with z-DEVD-fmk (35 μM) or z-VAD-fmk (35 μM), followed by PQR620 (300 nM) treatment. Cells were cultured for applied time periods, with cell apoptosis and viability tested by apoptotic nuclei staining **(G)** and CCK-8 **(H)** assays, respectively. Data were presented as mean ± standard deviation (SD, n=5). *p< 0.05 vs. “Veh” cells. **^#^** *p*< 0.05 vs. DMSO group **(G**, **H)**. “n.s”, non-statistical difference **(L**, **M)**. Experiments were repeated five times with similar results. Scale bar= 100 μm **(D**, **E)**.

We tested the potential effect of PQR620 on cell apoptosis. In pNSCLC-1 cells, the caspase-3 activity increased over eight folds after PQR620 (300 nM, 24 h) treatment (**Figure 2A**). Cleavages of caspase-3, poly(ADP-ribose) polymerase (PARP) and caspase-9 were detected in PQR620-treated pNSCLC-1 cells (**Figure 2B**), where ssDNA accumulation was detected (indicating DNA breaks, **Figure 2C**). JC-1 green monomers were accumulated in mitochondria of PQR620-treated pNSCLC-1 cells, suggesting mitochondrial depolarization (**Figure 2D**).

To further confirm cell apoptosis, we counted the number of apoptotic nuclei, which was displayed by condensed/fragmented Hoechst-33342 staining. Some cells were positive for TUNEL staining (purple) (**Figure 2E**). As demonstrated, the ratio of apoptotic nuclei (% vs. total nuclei) was significantly increased following PQR620 (300 nM, 48 h) treatment in pNSCLC-1 cells (**Figure 2E**). In addition, an increased number of Annexin V-positive cells (gated by FACS) further confirmed apoptosis activation by PQR620 (**Figure 2F**). Importantly, the caspase-3 inhibitor z-DEVD-fmk and the pan caspase inhibitor z-VAD-fmk almost blocked PQR620-induced apoptosis activation (apoptotic nuclei staining assay, **Figure 2G**) and viability (CCK-8 OD) reduction (**Figure 2H**) in pNSCLC-1 cells. These results indicated that caspase-apoptosis activation mediated PQR620-induced cytotoxicity in pNSCLC-1 cells.

In other primary (pNSCLC-2 and pNSCLC-3) and established (A549 and NCI-H1944) NSCLC cells, treatment with PQR620 (300 nM) induced caspase-3 activation (**Figure 2I**), mitochondrial depolarization (JC-1 green monomers intensity increase, **Figure 2J**), and increased ratio of apoptotic nuclei (**Figure 2K**), confirming apoptosis activation. In contrast, in primary lung epithelial cells (“pEpi”) and established BEAS-2B cells, same PQR620 (300 nM) treatment failed to significantly inhibit cell viability (CCK-8 OD, **Figure 2L**) and induce apoptosis activation (**Figure 2M**), suggesting a cancer cell-specific activity by PQR620.

### PQR620 Blocks mTOR Activation in NSCLC Cells

**Figure 3:**
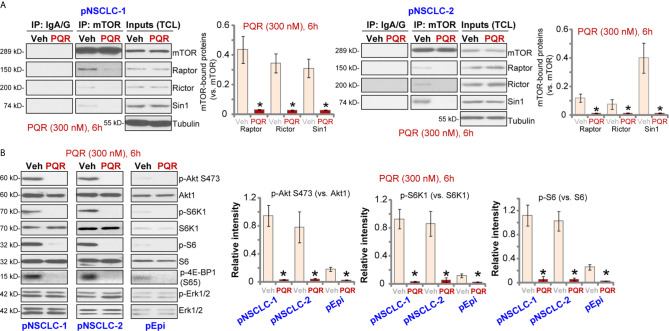
PQR620 blocks mTOR activation in NSCLC cells. Primary NSCLC cells, pNSCLC-1/-2, or primary lung epithelial cells (“pEpi”) were treated with PQR620 (300 nM) or the vehicle control (“Veh”), cells were further cultured for 6 h, mTOR immunoprecipitation with Rictor, Raptor and Sin1 were tested by co-immunoprecipitation assays **(A)** Expression of listed proteins was tested by Western blotting analyses (**A**: Inputs, and **B**). Quantification of the listed proteins was through the ImageJ software. Data were presented as mean ± standard deviation (SD, n=5). *p< 0.05 vs. “Veh” cells. Experiments were repeated five times with similar results obtained.

PQR620 is a novel and potent mTOR kinase inhibitor (16–18), we therefore tested its activity on mTOR activation in NSCLC cells. The co-immunoprecipitation (Co-IP) assay was performed to test mTOR assembles. As shown, in vehicle-treated pNSCLC-1 and pNSCLC-2 cells, mTOR immunoprecipitated with Rictor, Raptor and Sin1, showing integrated mTORC1 and mTORC2 complexes (9, 32, 33) (**Figure 3A**). Following PQR620 treatment, assembles of mTORC1 (mTOR-Raptor) and mTORC2 (mTOR-Rictor-Sin1) were disrupted in pNSCLC-1 and pNSCLC-2 cells (**Figure 3A**). Expression of mTOR, Raptor, Rictor, and Sin1 was unchanged (**Figure 3A**, “Inputs”, total cell lysates/”TCL”).

In pNSCLC-1 and pNSCLC-2 cells, PQR620 (300 nM) treatment almost blocked phosphorylation of Akt (Ser-473), the indicator of mTORC2 activation (**Figure 3B**). In addition, phosphorylations of S6K1 (Thr-389), S6 (Ser-235/236), and 4E-BP1 (Ser-65), indicators of mTORC1 activation, were largely inhibited as well (**Figure 3B**). Quantitative analyses confirmed that PQR620-induced inhibitions on Akt, S6K1, and S6 phosphorylations were significant (**Figure 3B**, right panels). Expressions and phosphorylation of Akt, S6K1, and S6, as well as Erk1/2 were not significantly affected by PQR620 in pNSCLC-1 or pNSCLC-2 cells (**Figure 3B**). Importantly, in primary lung epithelial cells basal phosphorylations of Akt, S6K1, and S6 were significantly lower than those in NSCLC cells (**Figure 3B**). This could explain the ineffectiveness of this compound in epithelial cells.

### PQR620-Induced Anti-NSCLC Cell Activity Is Not Solely Dependent on Akt-mTOR Inhibition

**Figure 4:**
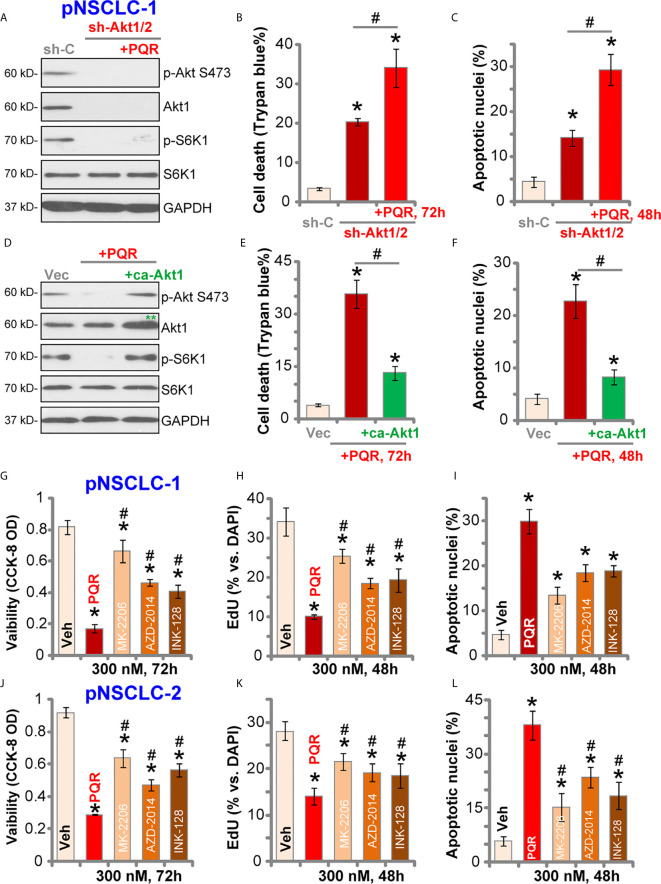
PQR620-induced anti-NSCLC cell activity is not solely dependent on Akt-mTOR inhibition. Stable pNSCLC-1 cells expressing Akt1/2 shRNA (sh-Akt1/2) were treated with or without PQR620 (“PQR”, 300 nM), and control cells were treated with scramble control shRNA (sh-C). Cells were further cultured for applied time periods, and expression of listed proteins was shown **(A)** Cell death and apoptosis were tested by Trypan blue **(B)** and apoptotic nuclei **(C)** staining assays, respectively. Stable pNSCLC-1 cells expressing constitutively-active Akt1 (ca-Akt1, S473D) were treated with or without PQR620 (“PQR”, 300 nM), and control cells were transfected with empty vector (Vec). Cells were further cultured for applied time periods, and expression of listed proteins was shown **(D)** Cell death **(E)** and apoptosis **(F)** were tested similarly. pNSCLC-1 **(G–I)** and pNSCLC-2 cells **(J**–**L)** were treated with 300 nM of PQR620 (“PQR”), MK-2206, AZD-2014 or INK-128 for applied time periods. Cell viability, proliferation, and apoptosis were tested by CCK-8 assay **(G**, **J)**, nuclear EdU staining **(H**, **K)**, and apoptotic nuclei staining **(I**, **L)** assays, respectively. Data were presented as mean ± standard deviation (SD, n=5). *p< 0.05 vs. “shC”/”Vec”/”Veh” cells. ^#^ *p*< 0.05 **(B**, **C**, **E**, **F)**. **^#^** *p*< 0.05 vs. PQR620 treatment **(G**–**L)**. Experiments were repeated five times with similar results obtained.

To test whether Akt-mTOR blockage is the primary reason of PQR620-induced anti-NSCLC cell activity, Akt1/2 shRNA lentiviral particles were transduced to pNSCLC-1 cells. Stable cells were established with selection by puromycin (“sh-Akt1/2” cells) where Akt expression was silenced (**Figure 4A**). Phosphorylation of Akt and S6K1 was completely blocked (**Figure 4A**). As shown, shRNA-induced silencing of Akt1/2 led to pNSCLC-1 cell death (Trypan blue ratio increase, **Figure 4B**) and apoptosis (apoptotic nuclei ratio increase, **Figure 4C**). Importantly, in sh-Akt1/2 cells, PQR620 (300 nM) was still able to induce cell death (**Figure 4B**) and apoptosis (**Figure 4C**). These results implied that Akt-mTOR-independent mechanisms could also be responsible for PQR620-induced cytotoxicity in pNSCLC-1 cells.

To further support our hypothesis, a constitutively-active Akt1 (ca-Akt1, S473D) was stably transfected to pNSCLC-1 cells. As shown, ca-Akt1 (labeled with double green stars, **Figure 4D**) completely restored phosphorylations of Akt and S6K1 in PQR620-treated pNSCLC-1 cells (**Figure 4D**). However, PQR620-induced pNSCLC-1 cell death (**Figure 4E**) and apoptosis (**Figure 4F**) were only partially inhibited by ca-Akt1. Moreover, in pNSCLC-1 and pNSCLC-2 cells, PQR620-induced viability (CCK-8 OD) reduction (**Figures 4G, J**), proliferation inhibition (EdU assays, **Figures 4H, K**) and apoptosis (apoptotic nuclei assays, **Figures 4I, L**) were significantly more potent than the known Akt-mTOR inhibitors, including the Akt specific inhibitor MK-2206 (34, 35) and two mTOR kinase inhibitors, AZD-2014 (36) and INK-128 (37). All inhibitors were utilized at same concentration (300 nM) (**Figures 4G–L**).

### PQR620 Inhibits SphK1 Activity and Induces Oxidative Injury in NSCLC Cells

**Figure 5:**
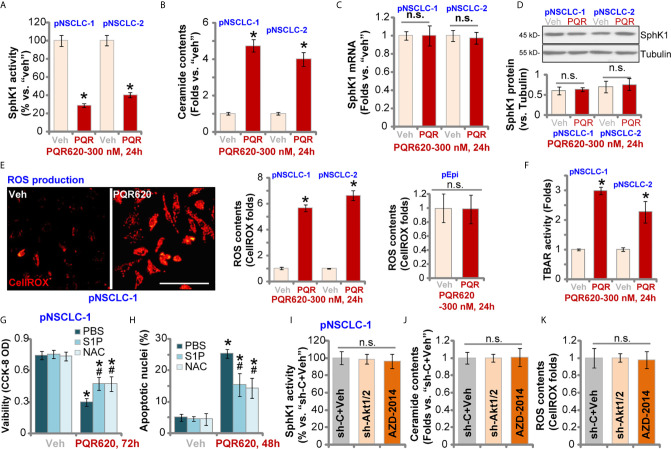
PQR620 inhibits SphK1 activity and induces oxidative injury in NSCLC cells. Primary NSCLC cells, pNSCLC-1/-2, or lung epithelial cells (“pEpi”), were treated with PQR620 (300 nM) or the vehicle control (“Veh”).Cells were further cultured for indicated time periods, and SphK1 activity **(A)**, ceramide contents **(B)**, *SphK1* mRNA **(C)** and protein **(D)** expression, as well as cellular ROS contents (by testing CellROX intensity, **E**) and lipid peroxidation (by testing TBAR activity, **F**) were tested using the described methods, with results normalized. pNSCLC-1 cells were treated with PQR620 (300 nM) first, then with or without sphingosine-1 phosphate (S1P, 1 μM) or NAC (400 μM). Cells were further cultured, with cell viability and apoptosis tested by CCK-8 **(G)** and apoptotic nuclei staining **(H)** assays, respectively. Relative SphK1 activity **(I)**, ceramide levels **(J)** and ROS contents **(K)** in pNSCLC-1 cells with Akt1/2 shRNA (sh-Akt1/2) are shown here with scramble control shRNA (sh-C) or AZD-2014 treatment (300 nM, 24 h). Data were presented as mean ± standard deviation (SD, n=5). *p< 0.05 vs. “Veh” cells. **^#^** *p*< 0.05 vs. PQR620 only treatment **(G**, **H)**. “n.s.” stands for non-statistical difference **(D**, **E**, **I**–**K)**. Experiments were repeated five times with similar results obtained. Scale Bar= 100 µm **(E)**.

Studies have shown that SphK1, which is essential for cancer cell survival, apoptosis resistance and migration, is overexpressed and/or hyper-activated in NSCLC (38–41). SphK1 inhibition or silencing should induce pro-apoptotic ceramide accumulation and NSCLC cell apoptosis (38–41). Here in pNSCLC-1 and pNSCLC-2 cells, SphK1 activity was robustly decreased after PQR620 treatment (**Figure 5A**). Consequently, cellular ceramide contents were increased (**Figure 5B**). *SphK1* mRNA (**Figure 5C**) and protein (**Figure 5D**) expression was however unchanged with PQR620 treatment.

When facing various stresses, robust ROS production and oxidative injury could induce NSCLC cell apoptosis. We therefore tested ROS levels in PQR620-treated NSCLC cells. In pNSCLC-1 and pNSCLC-2 cells, CellROX fluorescent intensity was robustly increased after PQR620 treatment, indicating ROS production (**Figure 5E**). In lung epithelial cells (“pEpi”), PQR620 failed to induce significant ROS production, as the CellROX intensity was unchanged (**Figure 5E**). The profound lipid peroxidation, evidenced by the increased TBAR activity, was detected in NSCLC cells after PQR620 stimulation (**Figure 5F**). These results implied that PQR620 induced ROS production and oxidative injury in NSCLC cells.

Experiments were carried out to examine whether SphK1 inhibition and ROS production were involved in PQR620-induced cytotoxicity in NSCLC cells. Sphingosine-1 phosphate (S1P), the anti-ceramide sphingolipid, as well as the antioxidant NAC were utilized. As shown, co-treatment with S1P or NAC could partially ameliorated PQR620-induced cell viability reduction (**Figure 5G**) and apoptosis **(** **Figure 5H**) in pNSCLC-1 cells. These results implied that SphK1 inhibition and ROS production contributed to PQR620-induced NSCLC cell death. Importantly, mTOR inactivation by Akt1/2 shRNA (see **Figure 4**) and AZD-2014 (300 nM, 24 h) treatment failed to significantly alter SphK1 activity (**Figure 5I**), ceramide contents (**Figure 5J**) or ROS production (**Figure 5K**). These results indicated that SphK1 inhibition and ROS production were unique actions by PQR620 in NSCLC cells, independent of mTOR inhibition. This could also explain the superior anti-NSCLC cell activity by this compound.

### PQR620 Administration Inhibits NSCLC Xenograft Growth in SCID Mice

**Figure 6:**
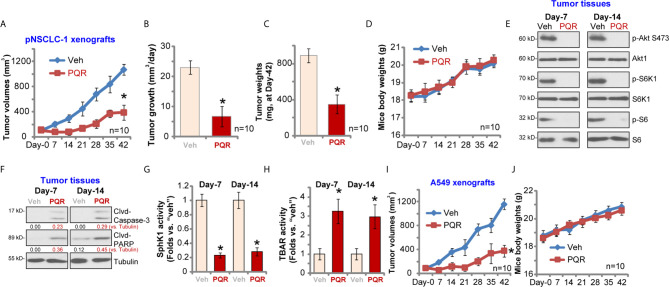
PQR620 administration inhibits NSCLC xenograft growth in SCID mice. SCID mice bearing pNSCLC-1 xenograft tumors **(A–H)** or A549 xenograft tumors **(I**, **J)** were administrated with PQR620 (oral gavage, 30 mg/kg, daily for 21 days, “PQR”) or vehicle control (“Veh”). Tumor volumes **(A**, **I)** and mice body weights **(D**, **J)** were recorded at every seven days for a total of 42 days. For pNSCLC-1 xenografts, the estimated daily tumor growth, in mm^3^ per day, was calculated by the described formula **(B)**; At day-42, pNSCLC-1 xenografts were individually isolated and weighted **(C)**. Expression of listed proteins in pNSCLC-1 xenograft tumor tissues was tested by Western blotting assays **(E**, **F)**; Relative SphK1 activity **(G)** and TBAR activity **(H)** were tested as well, with results normalized. Data were presented as mean ± standard deviation (SD). Ten mice were in each group (n = 10) **(A–D**, **I**, **J)**. *p< 0.05 vs. “Veh” group.

To study the potential effect of PQR620 *in vivo*, pNSCLC-1 cells were *s.c.* injected to flanks of SCID mice. Within three weeks, pNSCLC-1 xenografts were established (tumor volume close to 100 mm^3^, labeled as “Day-0”). Mice were then randomly assigned into two groups with 10 mice per group. Treatment group received PQR620 oral administration (“PQR,” 30 mg/kg, daily for 21 days). Control group were treated with vehicle control (“Veh”). Tumor growth curve results in **Figure 6A** demonstrated that PQR620 administration robustly inhibited pNSCLC-1 xenograft growth in SCID mice. We calculated the estimated daily tumor growth using the formula: (Tumor volume at Day-42—Tumor volume at Day-0)/42. Results again showed that PQR620 potently inhibited pNSCLC-1 xenograft growth *in vivo* (**Figure 6B**). At the end of experiments (Day-42), all tumors were isolated and weighted individually. As shown, pNSCLC-1 xenografts from PQR620-treated mice were significantly lighter than those with vehicle treatment (**Figure 6C**). Mice body weights were not significantly different between the two groups (**Figure 6D**). We failed to detect any apparent toxicities in mice.

Next, experiments were performed to examine whether PQR620 induced similar signaling changes *in vivo*. At Day-7 and Day-14, 3 h after initial PQR620/vehicle administration, one tumor of each group was isolated (total four tumor xenografts). Xenograft tissue lysates were obtained and subjected to Western blotting assays. Results in **Figure 6E** displayed that phosphorylations of S6K1-S6 and Akt (Ser-473) were almost completely blocked in PQR620-treated tumors. Contrarily, levels of cleaved caspase-3 and PARP were increased in pNSCLC-1 xenografts with PQR620 administration (**Figure 6F**), indicating apoptosis activation. Oral administration of PQR620 largely inhibited SphK1 activation in pNSCLC-1 xenograft tissues (**Figure 6G**). Furthermore, lipid peroxidation intensity was increased in pNSCLC-1 xenograft tissues with PQR620 treatment (**Figure 6H**), indicating significant oxidative injury. Therefore, PQR620 administration induced Akt-mTOR inactivation, apoptosis, SphK1 inhibition, and possible oxidative injury in pNSCLC-1 xenograft tissues.

Alternatively, A549 NSCLC cells were inoculated *via s.c.* injection to flanks of SCID mice. Xenograft tumors were established again within three weeks. As shown, oral administration of PQR620 largely inhibited A549 xenograft growth in SCID mice (**Figure 6I**). The mice body weights were again unchanged (**Figure 6J**). Together, these results suggested that PQR620 oral administration inhibited NSCLC xenograft growth in SCID mice.

## Discussion

Due to gene mutation, overexpression, and posttranslational modifications, aberrant mTOR activation is often detected in a significant proportion of NSCLC, which is heavily implicated in tumorigenesis and cancer progression (5). Increased mTOR expression and phosphorylation were observed in close to 90% of NSCLC patients with adenocarcinoma, while 60% of patients had large cell carcinoma and 40% of patients had squamous cell carcinoma (42–44). Thus, mTOR is an important therapeutic target of NSCLC.

A number of mTOR specific inhibitors are currently under preclinical investigations and in early phase of clinical trials for the treatment of NSCLC. The second generation of mTOR inhibitors have been developed as well (13, 14). Unlike the traditional mTOR inhibitors (rapamycin and its analogs), these agents are able to block both mTORC1 and mTORC2, and have pan-PI3K inhibitory activity (13, 14). One of this agent, BEZ235, is currently under phase I/II clinical trials. Early preclinical studies have demonstrated its potent activity against lung cancer (45). XL765 is another second generation mTOR kinase inhibitor and is being tested in a phase I trial in combination with erlotinib in NSCLC (46).

The results of this study suggested that PQR620 was able to exert potent anti-NSCLC cell activity. In primary NSCLC cells and established cell lines, PQR620 potently inhibited cell growth, proliferation and cell cycle progression, as well as cell migration and invasion. PQR620 provoked significant apoptosis activation in NSCLC cells. *In vivo*, oral administration of a single dose of PQR620 robustly inhibited NSCLC xenograft growth in SCID mice. SCID mice with PQR620 administration did not present any apparent toxicities. Therefore, PQR620 potently inhibited NSCLC cell growth.

Although PQR620 disrupted assembles of mTORC1 (mTOR-Raptor) and mTORC2 (mTOR-Rictor-Sin1), and inhibited Akt-S6K1-S6phosphorylations in NSCLC cells, our results suggested that mTOR-independent mechanisms also participated in PQR620-induced NSCLC cytotoxicity. First, PQR620-induced NSCLC cell death was significantly more potent than other known Akt-mTOR inhibitors (MK-2206, AZD-2014 and INK-128). Second, after restoring mTOR activation by caAkt1, we found only partially ameliorated PQR620-induced cytotoxicity in NSCLC cells. Third, in Akt-silenced NSCLC cells where mTOR activation was completely blocked, PQR620 was still able to induce cytotoxicity. Indeed, we found that SphK1 inhibition and ROS production participated in PQR620-induced NSCLC cell death.

SphK1 is therefore a potential oncotarget of NSCLC (38, 39). Ma et al. reported that SphK1 mediated signal transducer and activator of transcription 3 (STAT3) while promoting NSCLC cell proliferation and migration (47). In addition, Ni et al., showed that SphK1 is important for epithelial mesenchymal transition (EMT) in A549 cells (48). In the current study, we showed that PQR620 inhibited SphK1 and induced pro-apoptotic ceramide accumulation in primary NSCLC cells. Furthermore, SphK1 inhibition was detected in PQR620-treated NSCLC xenograft tissues. Conversely, S1P was able to attenuate PQR620-induced NSCLC cell death. SphK1 inactivation by PQR620 appeared to be mTOR-independent, as Akt1/2 shRNA and AZD-2014 failed to inhibit SphK1 activation in NSCLC cells. These results implied that concurrent inhibition of SphK1 could be an important mechanism to explain PQR620-induced superior anti-NSCLC cell activity.

A number of anti-cancer agents could induce ROS production and oxidative injury in NSCLC cells to cause cell apoptosis (49–51). Contrarily, antioxidants and ROS-scavenging strategies protected NSCLC cells from a number of anti-cancer agents (49–51). In the present study, we show that ROS production was significantly increased in PQR620-treated NSCLC cells. It was independent of mTOR inhibition, as ROS levels were unchanged in NSCLC cells with Akt1/2 shRNA and AZD-2014 treatment. Oxidative injury was also detected in NSCLC xenograft tissues with PQR620 administration. Importantly, NAC alleviated PQR620-induced apoptosis in NSCLC cells. Thus, PQR620-induced oxidative injury in NSCLC cells could be another reason to explain its superior anti-NSCLC cell activity.

## Conclusion

Development of new therapeutic agents for NSCLC is needed (52, 53). Here we found that PQR620 targeted multiple cascades (Akt-mTOR, SphK1 and ROS) and robustly suppressed NSCLC cell growth. PQR620 could be a promising and novel anti-NSCLC agent.

## Data Availability Statement

The original contributions presented in the study are included in the article/supplementary material. Further inquiries can be directed to the corresponding authors.

## Ethics Statement

The animal study was reviewed and approved by Animal Ethics Board of Nanchang University.

## Author Contributions

All the listed authors in the study carried out the experiments, participated in the design of the study and performed the statistical analysis, conceived of the study, and helped to draft the manuscript. All authors contributed to the article and approved the submitted version.

## Funding

Supported by the National Natural Science Foundation of China (81660391), Natural Science Foundation of Jiangxi Province (20202BABL206088), and by Special Fund for Postgraduate Innovation of Jiangxi Province (YC2020-B056).

## Conflict of Interest

The authors declare that the research was conducted in the absence of any commercial or financial relationships that could be construed as a potential conflict of interest.

The reviewer CC declared a shared affiliation with one of the authors WX to the handling editor at the time of the review.
